# BUILDING RESILIENCE USING PHOTOVOICE AMONG FAMILY CAREGIVERS

**DOI:** 10.1093/geroni/igad104.2973

**Published:** 2023-12-21

**Authors:** Mary Dioise Ramos

**Affiliations:** Kennesaw State University, Kennesaw, Georgia, United States

## Abstract

Filipino Americans remain one of the most underrepresented groups in research. It is challenging to identify the healthcare needs related to palliative and end-of-life care, especially among family caregivers. Palliative care is not yet well known in general, including among Filipino immigrants. Most people seem to adjust to the caregiver role and recover from Trauma/Emotional strain. However, some experience a long-lasting decline in their mental and physical health. This study will fill the gap in our understanding of the experiences of bereaved Filipino American family caregivers who have provided palliative and end-of-life care to their loved ones. This study aimed to explore the opportunities and challenges faced by Filipino American family caregivers who provided palliative and end-of-life care to their loved ones in the United States. The study also examined the impact of Photovoice as an intervention to build resilience, social support, and health-related quality of life among caregivers. The findings revealed that caregivers engaged in self-care activities, sought support from their social networks and healthcare providers, used coping strategies to manage emotional stress, found meaning in their caregiving experiences, and faced physical and emotional demands associated with caregiving. The Photovoice intervention was found to improve caregivers’ resilience and HRQOL significantly, but not their social support. Caregivers need assistance to manage the demands of providing care, including preparing for the future, securing help, and navigating agencies and relationships. Healthcare providers need to provide adequate support and resources to help caregivers manage the physical and emotional demands associated with caregiving.

